# Supplementary material for: Population Structure, Stratification, and Introgression of Human Structural Variation
Source: Cell. 2020 Jul 9;182(1):189–199.e15. doi: 10.1016/j.cell.2020.05.024 (PMC7369638; doi:10.1016/j.cell.2020.05.024)
Supplement: Table S1. Number of Samples per Population Analyzed in this Study (Passing QC), Stratified by Library Preparation and Sequencing Location, Related to Figure 1 — Total 911 samples from 54 populations. [file mmc1.docx]

| **Population** | **Region** | **sangerPCR** | **sangerPCRfree** | **sgdpPCR** | **sgdpPCRfree** | **Total** |
| --- | --- | --- | --- | --- | --- | --- |
| Adygei | EUROPE | 3 | 11 | 0 | 2 | 16 |
| Balochi | CENTRAL_SOUTH_ASIA | 3 | 19 | 0 | 2 | 24 |
| BantuKenya | AFRICA | 9 | 0 | 0 | 2 | 11 |
| BantuSouthAfrica | AFRICA | 3 | 1 | 0 | 4 | 8 |
| Basque | EUROPE | 0 | 21 | 0 | 2 | 23 |
| Bedouin | MIDDLE_EAST | 2 | 41 | 0 | 2 | 45 |
| BergamoItalian | EUROPE | 0 | 8 | 0 | 2 | 10 |
| Biaka | AFRICA | 15 | 3 | 0 | 2 | 20 |
| Bougainville | OCEANIA | 4 | 5 | 0 | 2 | 11 |
| Brahui | CENTRAL_SOUTH_ASIA | 3 | 20 | 0 | 2 | 25 |
| Burusho | CENTRAL_SOUTH_ASIA | 3 | 19 | 0 | 2 | 24 |
| Cambodian | EAST_ASIA | 3 | 4 | 0 | 2 | 9 |
| Colombian | AMERICA | 2 | 3 | 0 | 2 | 7 |
| Dai | EAST_ASIA | 0 | 4 | 1 | 3 | 8 |
| Daur | EAST_ASIA | 3 | 5 | 0 | 1 | 9 |
| Druze | MIDDLE_EAST | 3 | 37 | 0 | 2 | 42 |
| French | EUROPE | 0 | 24 | 1 | 2 | 27 |
| Han | EAST_ASIA | 0 | 29 | 1 | 2 | 32 |
| Hazara | CENTRAL_SOUTH_ASIA | 4 | 13 | 0 | 2 | 19 |
| Hezhen | EAST_ASIA | 0 | 7 | 0 | 2 | 9 |
| Japanese | EAST_ASIA | 0 | 25 | 0 | 2 | 27 |
| Kalash | CENTRAL_SOUTH_ASIA | 3 | 16 | 0 | 2 | 21 |
| Karitiana | AMERICA | 2 | 6 | 1 | 2 | 11 |
| Lahu | EAST_ASIA | 0 | 6 | 0 | 2 | 8 |
| Makrani | CENTRAL_SOUTH_ASIA | 3 | 20 | 0 | 2 | 25 |
| Mandenka | AFRICA | 12 | 5 | 1 | 2 | 20 |
| Maya | AMERICA | 2 | 17 | 0 | 2 | 21 |
| Mbuti | AFRICA | 7 | 1 | 1 | 3 | 12 |
| Miao | EAST_ASIA | 3 | 5 | 0 | 2 | 10 |
| Mongolian | EAST_ASIA | 2 | 5 | 0 | 2 | 9 |
| Mozabite | MIDDLE_EAST | 3 | 21 | 0 | 2 | 26 |
| Naxi | EAST_ASIA | 0 | 6 | 0 | 2 | 8 |
| NorthernHan | EAST_ASIA | 0 | 10 | 0 | 0 | 10 |
| Orcadian | EUROPE | 0 | 13 | 0 | 2 | 15 |
| Oroqen | EAST_ASIA | 0 | 6 | 0 | 2 | 8 |
| Palestinian | MIDDLE_EAST | 3 | 40 | 0 | 3 | 46 |
| PapuanHighlands | OCEANIA | 2 | 4 | 0 | 3 | 9 |
| PapuanSepik | OCEANIA | 5 | 2 | 0 | 1 | 8 |
| Pathan | CENTRAL_SOUTH_ASIA | 3 | 19 | 0 | 2 | 24 |
| Pima | AMERICA | 3 | 8 | 0 | 2 | 13 |
| Russian | EUROPE | 0 | 23 | 0 | 2 | 25 |
| San | AFRICA | 1 | 1 | 1 | 3 | 6 |
| Sardinian | EUROPE | 0 | 24 | 1 | 2 | 27 |
| She | EAST_ASIA | 0 | 8 | 0 | 2 | 10 |
| Sindhi | CENTRAL_SOUTH_ASIA | 3 | 19 | 0 | 2 | 24 |
| Surui | AMERICA | 3 | 3 | 0 | 2 | 8 |
| Tu | EAST_ASIA | 2 | 5 | 0 | 2 | 9 |
| Tujia | EAST_ASIA | 0 | 8 | 0 | 2 | 10 |
| Tuscan | EUROPE | 0 | 6 | 0 | 2 | 8 |
| Uygur | CENTRAL_SOUTH_ASIA | 3 | 5 | 0 | 2 | 10 |
| Xibo | EAST_ASIA | 3 | 4 | 0 | 2 | 9 |
| Yakut | EAST_ASIA | 3 | 20 | 0 | 2 | 25 |
| Yi | EAST_ASIA | 0 | 8 | 0 | 2 | 10 |
| Yoruba | AFRICA | 16 | 1 | 1 | 2 | 20 |

Table S1, Related to Figure 1: Number of samples per population analysed in this study (passing QC) stratified by library preparation and sequencing location. Total 911 samples from 54 populations.
