# Supplementary material for: Population Structure, Stratification, and Introgression of Human Structural Variation
Source: Cell. 2020 Jul 9;182(1):189–199.e15. doi: 10.1016/j.cell.2020.05.024 (PMC7369638; doi:10.1016/j.cell.2020.05.024)
Supplement: Table S2. Assembly Statistics and Number of Identified Non-Reference Unique Insertions per Sample, Related to Figure 4 [file mmc2.docx]

| **ID** | **Population** | **Assembly size (Mb)** | **ScaffN50 (Kb)** | **ContigN50 (Kb)** | **Raw_coverage** | **Number_NUI** |
| --- | --- | --- | --- | --- | --- | --- |
| HGDP00224 | Pathan | 2110 | 32.2 | 25.6 | 52.0x | 240 |
| HGDP00228 | Pathan | 2520 | 46.6 | 40.5 | 53.4x | 314 |
| HGDP00450 | Mbuti | 2710 | 234.9 | 71.5 | 51.6x | 523 |
| HGDP00460 | Biaka | 2760 | 761.5 | 102 | 52.1x | 609 |
| HGDP00472 | Biaka | 2460 | 45.6 | 38.2 | 45.9x | 347 |
| HGDP00542 | PapuanSepik | 2510 | 68.4 | 46.1 | 52.5x | 372 |
| HGDP00547 | PapuanSepik | 2510 | 48.4 | 40.6 | 42.4x | 344 |
| HGDP00549 | PapuanHighlands | 2350 | 46 | 33.3 | 52.4x | 260 |
| HGDP00551 | PapuanHighlands | 2460 | 64.3 | 43.5 | 40.2x | 363 |
| HGDP00562 | Druze | 2500 | 47.2 | 39.8 | 50.8x | 280 |
| HGDP00580 | Druze | 2730 | 303.7 | 77.3 | 46.8x | 470 |
| HGDP00670 | Sardinian | 2510 | 68.7 | 43.4 | 48.1x | 376 |
| HGDP00774 | Han | 2490 | 70.3 | 46.2 | 49.9x | 366 |
| HGDP00819 | Han | 2790 | 8330 | 142.6 | 52.4x | 597 |
| HGDP00930 | Yoruba | 2150 | 34.3 | 27.9 | 46.1x | 292 |
| HGDP00931 | Yoruba | 2410 | 46 | 38.3 | 48.4x | 330 |
| HGDP00946 | Yakut | 2040 | 30.9 | 25.5 | 47.7x | 205 |
| HGDP01013 | Karitiana | 2790 | 11160 | 137.4 | 51.1x | 574 |
| HGDP01019 | Karitiana | 2560 | 48.3 | 41.7 | 54.5x | 337 |
| HGDP01029 | San | 2100 | 42.1 | 28.2 | 45.7x | 288 |
| HGDP01032 | San | 2760 | 8480 | 115 | 48.9x | 711 |
| HGDP01043 | Pima | 2790 | 7370 | 141.2 | 50.9x | 599 |
| HGDP01056 | Pima | 2790 | 15440 | 119 | 54.0x | 463 |
| HGDP01067 | Sardinian | 2780 | 16310 | 141.5 | 52.4x | 638 |
| HGDP01081 | Mbuti | 2260 | 51 | 34.6 | 47.6x | 350 |

Table S2: Assembly statistics and number of identified NUIs per sample.
