# Supplementary material for: Population Structure, Stratification, and Introgression of Human Structural Variation
Source: Cell. 2020 Jul 9;182(1):189–199.e15. doi: 10.1016/j.cell.2020.05.024 (PMC7369638; doi:10.1016/j.cell.2020.05.024)
Supplement: Table S3. List of Clones that Mapped to the Location of the chr16p12 Oceania-Specific Duplication Shared with the Denisovan Genome, Related to Figure 2E — Probes were designed using the hg18 reference. Lifted over position in GRCh38 are provided. [file mmc3.docx]

| **Probe Name**  **in Figures** | **Probe ID** | **Chr16 Position (hg18)** | **Lifted over Position (GRCh38)** | **Note** |
| --- | --- | --- | --- | --- |
| C9 | WI2-1834C9 | 21465050-21506431 | chr16:21546228-21587609 | Original Site |
| D2 | WI2-431D2 | 22359681-22400825 | chr16:22440859-22482003 | 5' Reference (Also Segmental Duplication at insertion site) |
| K17 | WI2-555K17 | 22409260-22452683 | chr16:22490438-22533861 | 5' Reference (Also Segmental Duplication at insertion site) |
| N17 | WI2-1747N17 | 22464671-22508847 | chr16:22545849-22590025 | 5' Reference (Also Segmental Duplication at insertion site) |
| E20 | WI2-3914E20 | 22481967-22524149 | chr16:22563145-22605327 | Original Site |
| O10 | WI2-916O10 | 22526829-22568957 | chr16:22608007-22650135 | Original Site |
| F11 | WI2-810F11 | 22584142-22624353 | chr16:22665320-22705531 | Original Site |
| F15 | WI2-1829F15 | 22625840-22662216 | chr16:22707018-22743394 | Original Site |
| D19 | WI2-2529D19 | 22654207-22697236 | chr16:22735385-22778414 | Original Site |
| K21 | WI2-694K21 | 22727030-22769938 | chr16:22808208-22851116 | 3' Reference |
| N21 | RP11-368N21 | 29408699-29609853 | chr16:29489877-29691031 | Insertion site |
| J20 | WI2-2731J20 | 29369385-29408106 | chr16:29450563-29489284 | Insertion site |
| I24 | WI2-3063I24 | 29414050-29452497 | chr16:29495228-29533675 | Insertion site |
| G16 | WI2-1802G16 | 29446690-29485090 | chr16:29527868-29566268 | Insertion site |
| E19 | WI2-3518E19 | 29469634-29511102 | chr16:29550812-29592280 | Insertion site |
| O6 | WI2-0456O6 | 29510348-29548504 | chr16:29591526-29629682 | Insertion site |
| P22 | WI2-1399P22 | 29528833-29568651 | chr16:29610011-29649829 | Insertion site |
| K22 | WI2-2372K22 | 29559162-29602429 | chr16:29640340-29683607 | 3' Reference |
| I2 | RP11-504I2 | 29609848-29784210 | chr16:29691026-29865388 | 3' Reference |

Table S3: List of clones that mapped to the location of the chr16p12 Oceanian-specific duplication shared with the Denisovan genome. Probes were designed using the hg18 reference. Lifted over position in GRCh38 are provided.
